# Pilot study of physiotherapist-led versus music therapist-led breathing control exercises for young adults living with breathing pattern disorder: a randomised controlled trial protocol

**DOI:** 10.1136/bmjresp-2022-001414

**Published:** 2022-09-14

**Authors:** Adam Lewis, Elmar Kal, Claire Marie Nolan, Phoene Cave, Lizzie Grillo, Joy Conway, Mandy Jones

**Affiliations:** 1 Department of Health Sciences, Brunel University London, College of Health Medicine and Life Sciences, Uxbridge, UK; 2 Harefield Respiratory Research Group, Royal Brompton and Harefield Hospitals, London, UK; 3 National Heart and Lung Institute, Imperial College London, London, UK; 4 Physiotherapy Department, Royal Brompton and Harefield Hospitals, London, UK

**Keywords:** perception of asthma/breathlessness

## Abstract

**Introduction:**

Breathing pattern disorder (BPD) is an abnormal breathing pattern associated with biochemical, biomechanical and psychophysiological changes. While physiotherapy is often offered, limited evidence-based therapies for BPD are available. Music therapy-based singing exercises have been shown to improve quality of life for individuals with respiratory conditions and may also be beneficial for individuals living with BPD. No study has previously compared these participatory interventions in the treatment of people living with BPD.

**Methods and analysis:**

This is a study protocol for an assessor blinded 1:1 randomised controlled trial and qualitative interview study. Forty participants aged 18–40 years who score at least 19 on the Nijmegen Questionnaire (NQ) and do not have any underlying respiratory conditions will be recruited. Participants will be randomised to receive either physiotherapy-led or music therapy-led breathing exercises for 6 weeks. The primary outcome will be between-group difference in NQ post-intervention. Semistructured interviews with a purposive sample of participants will be performed. Qualitative data will be analysed using thematic analysis to better understand participants’ intervention and trial experiences.

**Ethics and dissemination:**

This study has received ethical approval by Brunel University London College of Health, Medicine and Life Science’s Research Ethics Committee (32483-MHR-Mar/2022-38624-3). The anonymised completed dataset will be made available as an open-access file via Brunel University London Figshare and the manuscript containing anonymised patient data will be published in an open-access journal.

**Trial registration number:**

This trial is registered on the Open Science Framework Registry (https://osf.io/u3ncw).

**What is already known on this topic**

- Breathing pattern disorders (BPDs) are prevalent in young adults with limited evidence-based therapy options available.

**What this study adds**

- This protocol details two interventions which will be compared for their effectiveness for the first time.
- Novel participant experiences of these interventions will also be explored.

**How this study might affect research, practice or policy**

- This protocol will establish interventions which can be reproduced in future studies.
- If both physiotherapy and music therapy are considered effective in treating BPD in the absence of underlying respiratory disease, this research could be considered for inclusion in future treatment guidelines.

## Introduction

Breathing pattern disorder (BPD), otherwise known as dysfunctional breathing (DB), is defined as having an abnormal breathing pattern according to alterations in breathing timing, coupling to movement, the passage of air in and out of the respiratory system, distribution of breathing and focus of attention.^1^ While there is ongoing discussion about the exact definition of what constitutes BPD, it is widely recognised that BPD is multifaceted, being associated with biochemical, biomechanical and/or psychophysiological changes.^2–6^ Specifically, regarding the biochemical dimension, people may experience disturbances in blood pH homeostasis. Biomechanical changes include alterations in respiratory mechanics, muscle tone, strength, coordination and the use of the nose or mouth for breathing. Finally, on a psychophysiological level, BPD seems to be associated with specific maladaptive thoughts, feelings, breathing behaviours and symptoms.^2^ Taken together, BPD includes both subjective and objective breathing pattern changes, and can therefore substantially impacts a person’s quality of life.^7–13^ However, not all BPDs combine all biochemical, biomechanical and psychophysiological changes. Estimates of BPD’s prevalence vary, but it has been reported to occur in 8% of adults with a mean age of 44^14^ to 22% in young adults with a mean age of 21,^12^ while secondary (ie, due to underlying pathology) BPD may affect up to a third of adults with asthma.^14^ Outcomes used to measure BPD include cardiopulmonary exercise testing,^15^ the Manual Assessment of Respiratory Motion (MARM),^16^ the Self-Evaluation of Breathing Questionnaire (SEBQ),^17^ the Nijmegen Questionnaire (NQ)^1 10 18^ and the Breathing Pattern Assessment Tool (BPAT).^11^ Neither the NQ or BPAT outcomes have established minimal clinical important differences related to the comparison of interventions but are commonly used and combine inexpensive participant and assessor analysis of breathing pattern subjectively and objectively.

Limited evidence-based therapies for BPD are available. Current standard care is the provision of breathing exercises delivered by a physiotherapist. Evidence shows that clinical benefits are achieved with physiotherapist-led breathing exercises for individuals with secondary BPD.^9 19–21^ However, a high-quality evidence for these interventions is limited,^22^ particularly for individuals with primary dysfunctional breathing in the absence of other pathology. Singing for Lung Heath (SLH) is an intervention designed to enable people with chronic respiratory disease to use singing exercises and repertoire to enable them to manage their symptoms.^23^ Music therapy simultaneously targets physiological and psychological changes to help improve control of breathing.^24–27^ SLH has been shown to be beneficial at improving the quality of life and perceived breathing control of patients with chronic obstructive pulmonary disease in a UK-wide service evaluation, and in studies with comparison groups receiving no active intervention, or alternative group social interaction in chronic obstructive pulmonary disease (COPD).^23 28–30^ Forty-seven per cent of individuals with COPD have secondary BPD.^31^ Singing interventions may also be applicable and beneficial for individuals living with primary BPD. Singing-based exercises have also been shown to help improve the mental health component of quality of life and perceived exertional breathlessness for those with persistent symptoms post COVID-19.^32^ The proposed pilot study will explore whether there are any significant differences in measures of breathing pattern and related patient-reported outcomes between physiotherapist-led breathing control exercises (as provided in ‘standard’ care) compared with music therapist-led breathing control exercises for individuals living with primary BPD. Data analysis will inform sample size calculations for a more definitive trial.

This manuscript has been prepared according to the Standard Protocol Items: Recommendations for Interventional Trials 2013 checklist.^33^

## Methods and analysis

### Population

We aim to recruit 40 young adults aged between 18 and 40 years who have BPD, using Brunel University London Research Participation website, posters placed around campus and via investigator and Brunel University affiliated social media accounts. This sample size is in line with previously recommended ranges for pilot and feasibility designs.^34 35^ This is an assessor blinded 1:1 randomised controlled trial (RCT) pilot study with qualitative interview substudy.

Inclusion criteria are as follows:

- Young adults between 18 and 40 years with a score of at least 4 on the BPAT^11^ or at least 19 on the NQ.^18^ BPDs are common in younger populations^12^ and we want to optimise the interventions based on findings in this study before testing in potentially more frail populations. Both outcomes will be used as there is no single gold standard diagnosis for BPD.
- Access to an internet-enabled device.
- Normal spirometry according to forced expiratory volume in the first second (FEV_1_) and forced vital capacity (FVC) above 80% of that predicted for age, height, sex and race.

Exclusion criteria are as follows:

1. Previously received one-to-one singing tuition or physiotherapy led breathing control exercises.
2. Previously or currently member of a choir.
3. Diagnosis of other respiratory diseases which may lead to BPD (i.e., Asthma, COPD, long COVID, Bronchiectasis, Interstitial Lung Disease, chronic cough, sinus disease).
4. Diagnosis of neurological disease which impacts on respiratory system.
5. Unable to provide consent.
6. Pregnant

## Recruitment

### Online screening

Participants who register their interest to participate in the study will be sent an email asking them to complete the NQ,^10^ a patient-reported outcome measure that assesses functional respiratory complaints.^1 24^ The NQ has become the standard outcome measure used to identify BPD. Only participants who score at least 19 at screening^1 18^ will continue onto the study and repeat the questionnaire at their baseline assessment and post therapy.

### Consent

Face-to-face informed consent will be obtained prior to any study assessment or intervention taking place. The investigator or designee will explain that the participants are under no obligation to enter the trial and that they can withdraw at any time during the trial, without having to give a reason. A copy of the signed informed consent form will be given to the study participant. The original signed consent form will only be accessible to members of the study staff.

### Face-to-face baseline/screening assessment

Assessments will be performed by an individual who has received training in breathing pattern assessment. The assessment will include the following:

Medical history, medication history, social history and demographic information such as age, sex, height and weight will be recorded. Next, screening and baseline spirometry will be performed using a portable spirometer (Micromedical Microlab 3500 Mk8) according to international guidelines.^36^ Should any abnormal finding become present following the spirometry, that is, FEV_1_ or FVC under 80% predicted, repeat spirometry may be indicated. Should abnormal findings be repeated individuals, will not continue into the study and the participant will be encouraged to contact their General Practitioner.

The participant will then complete the following outcomes:

The Nijmegen Questionnaire as described above.

The Breathing Hypervigilance Questionnaire (Breathe-VQ) is a six-question Likert scale response questionnaire. It has been developed by the current study team and has shown to have good construct validity, internal consistency and test-retest reliability in a study comprising of young dysfunctional breathers and non-dysfunctional breathers.^37^ The Breathe-VQ screens for the presence of excessive anxious, conscious monitoring of breathing state (‘hypervigilance’), which is thought to potentially contribute to the experience of breathlessness.^38^ Our hypothesis is that the interventions could also indirectly help to improve breathing pattern by reducing such vigilance and awareness. The Breathe-VQ will be used as a secondary outcome measure exploring its responsiveness to change post intervention for the first time.

The Breathing Pattern Assessment Tool: This is a clinician-led assessment of BPD.^11^ The clinician scores between 0 and 2 for different areas of assessing breathing pattern, including abdominal/chest movement, inspiratory flow, expiratory flow, channel of inspiration (nose or mouth), air hunger, respiratory rate and rhythm. The higher the score, the worse the breathing pattern. A score of at least 4 indicates BPD. Individuals will be asked which time of year their breathing is worse, and if there are any significant triggers for their BPD.

Participants who score at least 19 on the NQ or 4 on the BPAT will continue onto the study. Participants who do not record a score of at least 19 on the NQ or 4 on the BPAT will be excluded from the study at this stage. This information will be important to plan for a future definitive study. Participants will complete the Breathe-VQ, followed by the NQ, and a trained research assistant will then perform a BPAT in that order.

In total, the baseline screening/assessment will last approximately 1 hour.

### Randomisation

Following the baseline screening/initial assessment, individuals will be randomised on a 1:1 basis based on a computer-generated random sequence generated using random.org, either to music therapist-led breathing exercises or physiotherapy-led breathing exercises (the next subsection details these interventions). Randomisation will be performed by an independent member of staff not involved in baseline or follow-up assessments and confirmation of group allocation will be provided online. The assessor will be blinded to which group the participants have been randomised to. Participants will be instructed to not disclose which intervention arm they have been allocated to. Should their intervention become known to the assessor at any time, another researcher will perform the follow-up assessment. Due to the nature of the interventions, it is impossible to blind participants to the intervention.

### Intervention details

Participants will be randomly assigned to one of the following two interventions:

#### Intervention A

Music therapist-led breathing exercises: One-to-one face-to-face breathing exercises will be led by a music therapist (HCPCreg). Exercises will be based on previously established core components of SLH exercises which have established intervention fidelity and for which no serious adverse events have been reported in previous studies.^23 39^

#### Intervention B

Face-to-face physiotherapist-led breathing exercises: Unphonated breathing control exercises will be led by a physiotherapist and expert in BPD. Exercises will be performed according to best practice guidance.^1 21 40 41^

### Assessment part of the interventions (Session 1)

Both the physiotherapist-led intervention (Intervention A) and the music therapist-led intervention (Intervention B) include an assessment component from the physiotherapist and music therapist. Participants will return on a separate day within a month following their baseline assessment and receive an initial hour-long, face-to-face, one-to-one assessment and training session by a music therapist or physiotherapist. The therapist will check if nothing major changed in between the time of baseline appointment and screening session, in terms of their functional status, based on self-report. This initial training session will be done in line with contemporary university guidance and regulations in relation to COVID-19, which currently include using well-ventilated spaces and masks worn by assessment staff. Participants will need to take masks off during assessment and intervention. Following the first session, an individualised treatment plan will be recommended and home exercises given accordingly, aligned with the structure of each intervention method. Online supplemental table 1 provides a detailed overview of the two interventions.

10.1136/bmjresp-2022-001414.supp1 Supplementary data

### Physiotherapist and music therapist intervention introduction (Session 1)

For both interventions, during this session the focus will be on awareness and self-assessment of breathing pattern followed by the teaching of breathing exercises aimed at altering breathing pattern. Following this, the participants will be encouraged to perform a set of 5 min of home exercises, for 6 weeks, morning and evening every day, with advice and scope for progression or regression of minutes of practice and/or difficulty of tasks. A paper home exercise diary will be provided to the participant regardless of which group they are randomised to. This home exercise diary will be individually tailored according to progress made in the first face-to-face treatment session. This diary will include days of the week, exercises performed and minutes of practice per exercise.

### Refresher and progression follow-up (Session 2)

Each participant will receive one online one-to-one 30-minute follow-up session, 3 weeks after the initial training session (either provided by the music therapist or by the physiotherapist, depending on group allocation). This 30-minute session will offer repeat intervention and re-focus exercises to those which are most beneficial to the participant at that stage (as determined by the therapist) and can be progressed accordingly. Therapists will make notes of these meetings, to record their decision making and justification. We acknowledge that in clinical practice patients are often offered further follow-ups with progression to assessment of BPD during exercise. However, due to funding limitations for this pilot study, further assessments will not be possible. Evidence suggests that the majority of people with BPD do not require repeated follow-ups to gain significant clinical benefit.^1 42^

### Similarity of interventions

The interventions will be similar in terms of the dose provided and contact time with the respective therapist. However, the music therapist-led exercises will be predominantly phonated, whereas the physiotherapist-led breathing exercises will be unphonated. Both interventions may use bio-feedback, visualisation, relaxation-oriented strategies and counting strategies. The toolbox of options for therapy is standardised per intervention, but not every participant will receive all possible exercises. Therapists will record which exercises are given to each participant. The intervention will be individually tailored for the patient, which is common clinical practice. This also reflects other areas of exercise-based modalities, such as pulmonary rehabilitation, whereby a fixed set of possible exercises is available to choose from, but *what* exercise is chosen, and *how* that exercise is performed are individually tailored based on clinical judgement.^43^

### Follow-up assessment

Participants will be asked to return to Brunel University London, within a 2-week timeframe following their final week performing the intervention (so either in week 6 or week 7), to repeat all the measurements included at the screening assessment. Adverse events will also be recorded. Home exercise diaries will be collected during this assessment. As such, there are four participant contact points in total: (1) baseline screening/assessment; (2) training session 1, in week 1; (3) intermediate refresher session in week 3; (4) follow-up assessment in week 6 (or week 7). As stated earlier, the assessment will be performed by a blinded assessor.

### Qualitative study

Semistructured one-to-one interviews with a purposive sample of 21 participants will be performed in order to explore their perceptions of participating in the trial. We aim to recruit seven people randomised to the music therapist-led led breathing exercises, seven people randomised to the physiotherapist-led breathing exercises and (up to) seven non-completers of the interventions, defined as participants who did not participate in the half-way online meeting and/or who completed less than two thirds of the recommended exercise sessions (4 out of 6 weeks’ equivalent sessions). Interviews (and analysis) will be led by AL, who has extensive experience with qualitative research in the context of respiratory physiotherapy and will be completed via MS Teams. The topic guide (see appendices) includes questions about experiences of trial participation, attitudes towards content, delivery and timing of the intervention, and suggestions for improvements (both regarding the interventions as well as the larger study). It is expected that the qualitative interviews will last between 30 and 60 min. All interviews will be recorded using a Philips digital Dictaphone or MS Teams. Digital files will be uploaded to a secure online data repository, OneDrive, which all study team members involved in the analysis will have access to.

#### Primary outcome

- Between-group differences in change of NQ score post intervention (mean, SD, CIs and effect size).

#### Secondary outcomes

##### Feasibility

- Number of individuals completing both arms of the RCT study. At least 66% completion will support using that intervention in a future definitive randomised controlled study. The study team determined that below a 66% completion rate would either indicate significant changes to the study design are required prior to performing a definitive trial, or that a further definitive study is not feasible depending on other outcome analysis.
- Percentage of individuals recruited from screening, according to use of Nijmegen and BPAT screening tool.
- Perceived participant experiences of interventions and the feasibility of the study design, collected via semistructured interview.

##### Insight into effect of interventions

- Percentage of participants in each arm who are deemed recovered post-therapy (less than 17) and therefore no longer diagnosed with BPD.^41^
- Percentage of participants in each arm who demonstrate clinically relevant change in the NQ (at least 10 points).^41^
- Between-group differences in Breathe-VQ questionnaire.
- Between-group differences in BPAT scores.

### Statistical analyses

Forty participants is considered to be a suitable sample size for intervention pilot studies.^44^ This number of participants accounts for a likely small effect size between groups and an 80% powered definitive trial.^45^

For continuous outcome data from questionnaire data, data will be presented as mean (SD) or median (range). Comparisons will be made using a two-way repeated measures analysis of variance (ANOVA), with group as the between-participants factor and timepoint as the within-participants factor. This will be followed by post hoc t-tests if interaction is significant. Non-parametric data will be analysed using generalised estimation equation modelling. All tests will be two-sided and with alpha set at 0.05. Cohen’s *d* and *r* effect sizes will be calculated for parametric and non-parametric tests, respectively. We will also present a sample size calculation for a future definitive trial based on the primary outcome NQ results, and results regarding screening, recruitment and drop-out rates.

Additional exploratory analysis will be performed of between-group differences for the BPAT and Breathe-VQ due to the novelty of using these outcome measures in this population and because they may have important additional information on domains that are relevant to BPD.

No interim analyses are planned.

### Qualitative analysis

Qualitative interviews will be analysed using critical realist thematic analysis with a combination of a deductive process with prior knowledge of what is required for investigating feasibility of a future RCT and what processes occur within an RCT.^46^ Inductive analysis will enable a richer understanding of novel experiences of individuals receiving interventions which have no prior evidence-base for in their population.

### Patient and public involvement statement

Patients and the public were not involved in the design of this study. However, the qualitative interviews of participants who have BPD within this pilot study will directly inform the design of a future definitive trial following this pilot. For such a follow-up trial, the public and patients will be involved in the preparation (eg, planning of study, potential grant writing process), and costed patient and public involvement representatives will become members of the Trial Steering Committee.

### Safety reporting

Adverse events will be recorded and followed up until resolved.

Individuals who return a positive lateral flow test and subsequent PCR test at any stage after consent to the study will be withdrawn from continuing in the study due to the known respiratory complications arising from COVID-19.^47^

### Reporting urgent safety measures

The sponsor and/or the investigator may take appropriate urgent safety measures in order to protect participants against any immediate hazard to their health or safety.

The investigator will immediately and in any event no later than 3 days from the date the measures are taken, give written notice to the Research Ethics Committee (REC) and the study sponsor of the measures taken and the circumstances giving rise to those measures. In view of changing COVID-19 cases and restrictions, this may be necessary.

In order to prevent any delays in the reporting timelines, the sponsor has delegated this responsibility to the chief investigator/principal investigator (CI/PI). Therefore, the CI/PI must report any urgent safety measures to the REC directly, and in parallel to the sponsor.

### Data management and quality assurance

#### Confidentiality

All data will be handled in accordance with the Data Protection Act (2018), NHS Caldecott Principles, The UK Policy Framework for Health and Social Care Research and the condition of the REC approval.

The Case Report Forms (CRFs) will not bear the subject’s name or other personal identifiable data. The subject’s study identification number (ID) will be used for identification.

No data will be shared with any external organisation during the study without appropriate consent and data sharing agreement in place, as applicable. At the end of the study, the anonymised dataset will be made available as an open-access file via Brunel University London Figshare page and the manuscript containing anonymised patient data will be published in an open-access journal.

Due to funding limitations, there is no Data Monitoring Committee (DMC) for this pilot study. JC is providing academic oversight during the pilot study and will not be involved in primary data analysis. Brunel University London’s Research Support and Development Office will monitor the study. The inclusion of a DMC is planned for a future definitive study.

### Data handling and analysis

All CRF data will be inputted at Brunel University London Mary Seacole Building by study staff including chief investigator, who have not been involved in original data entry will provide quality assurance checks using the e-CRF on Microsoft Excel and paper-based records for accuracy and quality assurance within a monitoring role.

### Discontinuation

All participants are free to withdraw at any time from either intervention. Reasons for withdrawal will be collected and timing will be recorded because this information will help plan for the future definitive study. Withdrawn subjects will not be replaced on the trial.

### Monitoring and auditing

The study will be monitored and audited by the Brunel University Research Support and Development Office research officers.

### Direct access to source data

The investigator(s)/Brunel University London will permit study-related monitoring, audits, REC review and regulatory inspection(s), providing direct access to source data/documents. Study participants are informed of this during the informed consent discussion. Participants will consent to provide access to their notes.

### Insurance and indemnity

Brunel University London is liable for negligent harm to individuals covered by their duty of care. Brunel University London employing researchers are liable for negligent harm caused by the design of studies they initiate.
