# Supplementary material for: Pilot study of physiotherapist-led versus music therapist-led breathing control exercises for young adults living with breathing pattern disorder: a randomised controlled trial protocol
Source: BMJ Open Respir Res. 2022 Sep 14;9(1):e001414. doi: 10.1136/bmjresp-2022-001414 (PMC9476152; doi:10.1136/bmjresp-2022-001414)
Supplement: Supplementary data [file bmjresp-2022-001414supp001.pdf]

**Table 1 – Intervention details table**

| Component | Physiotherapy-Led | Music Therapist-Led |
|---|---|---|
| Assessment | SUBJECTIVE REPORT OF SYMPTONS<br>Patient description of Symptoms (Sx) *(record words used by patient to describe Sx)*<br>Patients own awareness of breathing pattern<br>Triggers to Sx<br>Recovery techniques / Easing factors<br>Air hunger signs *(yawning / sighing / clearing throat / tingling hands/feet)*<br><br>NASAL SYMPTOMS<br>Blocked or runny nose<br>Sinus pain<br>Postnasal Drip<br>Altered sense of smell<br><br>VOICE/Upper Airway<br>Voice changes *e.g., husky/strained/lost voice*<br>Closure/discomfort in throat<br><br>EXERCISE ABILITY<br>Frequency of exercise<br>Intensity of exercise<br>Time spent on exercise<br>Type of exercise<br>Sx with exercise<br>*-SOB/cough/airway closure*<br>General physical activity levels<br><br>SLEEP<br>Quality/duration<br><br>SOCIAL HISTORY<br>Family<br>Work<br>Hobbies | Discussion of client's self - observation around relationship to breath and issues, triggers, response.<br><br>SOCIAL HISTORY<br>Family<br>Work<br>Hobbies<br><br>PSYCHOLOGICAL HISTORY<br>History of psychological illness<br>Stress and coping mechanisms<br><br>**OBSERVATION OF BREATHING**<br>Mouth/Nose breathing<br>Upper/Lower chest<br>Respiratory Rate<br>Sounds on inspiration and expiration<br><br>Observation of client:<br>Sitting, standing, silent, walking and talking and still.<br><br>Talking about music.<br>Finding out client's interest and songs that have special meaning/memory.<br><br>Observe as client listens and talks. Observe difference in breath as they talk about their 'symptom picture' and how they talk about their favourite song. |

| | | |
|---|---|---|
| | PSYCHOLOGICAL HISTORY<br>History of psychological illness<br>Stress and coping mechanisms<br><br>**OBSERVATION OF BREATHING**<br>Mouth/Nose breathing<br>Upper/Lower chest<br>Respiratory Rate<br>Sounds on inspiration and expiration<br>Air hunger<br>Accessory muscle use<br>Rhythm of breathing<br>Inspiratory / Expiratory ratio<br>Breath Hold (possibly)<br>**ABDOMINAL/CORE Assessment**<br>-movement of diaphragm<br>-abdominal tension present<br>-core strength<br>-bradcliff angle<br><br>**VOICE**<br>*-upper airway sounds*<br>*- voice quality*<br><br>**COUGH**<br>*-nature*<br>*-type*<br>*-frequency*<br>Clearing throat<br><br>**EXERCISE / FUNCTIONAL SYMPTOMS**<br>(record method of assessment e.g., walk / formal test / stairs)<br>Changes to breathing pattern during Ax<br>-Work of breathing<br>-Accessory muscle use<br>-SpO2<br>-HR | |

| | | |
|---|---|---|
| | -Cough<br>- Audible inspiratory/expiratory sounds | |
| Warm-up | Not applicable for the physio assessment. | Introduce some physical warm-ups (use Singing for Breathing CD).<br><br>Notice where the breath is restricted and discuss any holding patterns. |
| Bio-feedback | Palpation during breathing and movement, using mirrors, to support with self assessment and awareness. | Voiced fricatives and self - palpation – sound and touch. |
| Awareness | Patient’s awareness of breathing. Sometimes patients don’t know what their complaint is until they have it explained by the physiotherapist. | Breath being responsive to the in-the-moment activity. Whole-body relationships promoted. |
| Posture | **POSTURAL ASSESSMENT**<br>Head position/ROM Cervical / Thoracic spine<br>Core stability | Relationship between feet and rest of body, hip flexors, psoas, back of the waist, back of the neck – what is happening with the head |

| | | |
|---|---|---|
| | MUSCLE ASSESSMENT<br>Accessory muscle activity<br><br>OBJECTIVE ASSESSMENTS<br>Bradcliff angle, ski jump | placement – observation to observe and bring awareness to holding patterns. |
| Self-assessment (patient education strategy) | Staged approached to breathing (self) assessment:<br>1) position<br>2) posture and muscle tension<br>3) nose v mouth breathing (sound/flow)<br>4) apical v abdominal movement (HiLo Test)<br>5) Respiratory rate<br>6) inspiratory and expiratory time and pause (Ratio) | 360 degree (or as far as possible) self-palpation of chest and abdomen. |
| Breathing control | Use self-assessment approach to breathing control.<br><br>Good position (start in supine, progress to half sitting, sitting, standing, movement).<br><br>Flow & sound, feeling of breath in and out nose (not mouth).<br><br>Where breath is happening (apical v abdomen).<br><br>Awareness of rate and rhythm of breath.<br><br>Increasing expiratory time, pause between breaths.<br><br>Additional options for Rx: manual overpressure of apical breath, gentle weight on abdomen (rolled socks, | Relational whole body.<br><br>Structural integration.<br><br>Appropriate to activity.<br><br>Gentle weight on belly.<br><br>Voiced and unvoiced.<br><br>Pitched and unpitched.<br><br>Move into floor work bring awareness to where body touches the floor. Start to observe the breath. Introduce accent method. Unvoiced. Voiced fricatives.<br><br>Move to side lying, seating and standing. |

| | | |
|---|---|---|
| | wheat bags or rice-filled socks) for biofeedback | |
| Functional assessment | How does breath change with walking / exercise / stairs.<br><br>How does rate change, are nose/mouth breathing utilised and at what point, upper and lower chest, expiratory time.<br>Recovery post exercise. | Is breath working responsively and appropriately to activity?<br><br>Offer physical movement practice appropriate to client and ask them to observe if breath use changed pre, during or after movement (breath holding or over breathing for example). |
| Muscle activity & palpation | Muscle tension.<br>Cervical spine and Thoracic spine mobility.<br>Bradcliff angle (ribs).<br>Abdominal tension.<br><br>See objective and posture sections | Not applicable for the music-therapist led intervention |
| Observation | General observation looking at how participants are holding themselves, body movements, posture. | Notice how the patient enters the room in term of posture, pace, standing, sitting - how is the person combining breath use with how they are moving and still – observe posture, what are the holding patterns? How have the skeleton and muscles been impacted – how effortful is their sitting, standing and moving – how can they be offered choices in more ease |
| Muscle stretching/work 1 | Stretching advice given to tight musculature which is thought to restrict optimum breathing pattern for the individual. | Upper body ie jaw, neck and upper chest, shoulders and even then would need to be relational with psoas, sacrum, hips and feet. |

| | | |
|---|---|---|
| Core-activation/muscle work 2 | Teach awareness of core activity and how this can impact on breathing.<br>Teach core stability (Transverse Abdominus) and importance of breathing during activation of these muscles. | Bring awareness and introduce exercises to engage the transversus abdominus and obliques. |
| Self-management | Use the volume analogy that different aspects of body function and movements will turn breathing 'up' and that this can impact on severity of symptoms. That Breathing Control can then be used to turn this volume back 'down' | How is the patient self-managing their condition? What are they using to help themselves and what is successful / not successful - what observations and reflections have they made. |
| Psychological component | Build understanding with participants regarding what other components could be influencing breathing.<br><br>Ensure the patient feels validated with their breathing as well as how their own 'stress/emotion' etc impacts on breathing.<br><br>Consider state of mental health and develop awareness of how emotion/tension can influence breathing. | A separate psychological component is not delivered. Rather the clinician integrates psychological management holistically in other components. |
| Use of voice | No vocal exercises performed within the physiotherapy-led intervention. | Unvoiced into voiced – discovery of the relationship between breath and phonation – Assessment of where the effort levels are (throat, chest, abdomen - how do the effort levels change as patient moves from lying to sidelying to sitting to standing to walking to dancing) |
| Song | Not applicable for the physiotherapy intervention. | Introducing song as both a focus and a distraction - talk |

| | | |
|---|---|---|
| | | about music - talk about song choices - think desert island discs - distracting from the moment but giving a space for a positive body memory - start to move from exercises to speech to song – observe/discuss what they notice when they sing utilising voiced fricatives and accent method and gestural movement with phrase lengths to reconnect body, breath and voice |
| Relaxation | Progressive muscle relaxation, guided imagery, changing posture. | Through music, Music Therapist led visualisation, relaxation within and through change of repertoire. |
